# Reversible cerebral vasoconstriction syndrome and posterior reversible encephalopathy syndrome following vaccination: analysis of the VAERS database and systematic review

**DOI:** 10.1097/MS9.0000000000001407

**Published:** 2023-10-18

**Authors:** Bahadar S. Srichawla, Ton Fang, Vincent Kipkorir, Maria A. Garcia-Dominguez

**Affiliations:** a Department of Neurology, University of Massachusetts, Chan Medical School, Massachusetts, USA; b Department of Medicine, University of Nairobi, Nairobi, Kenya

**Keywords:** posterior reversible encephalopathy syndrome, PRES, RCVS, reversible cerebral vasoconstriction syndrome, vaccination, vaccine

## Abstract

**Objectives:**

This study aimed to analyze the Vaccine Adverse Event Reporting System (VAERS) database and systematically review the literature to provide a comprehensive analysis of reversible cerebral vasoconstriction syndrome (RCVS) and posterior reversible encephalopathy syndrome (PRES) secondary to vaccination.

**Methods:**

The authors analyzed the VAERS database and conducted a systematic review following PRISMA guidelines. The inclusion criteria for VAERS data were a score of ≥3 on the RCVS_2_ score and/or radiographic findings consistent with the diagnosis of RCVS or PRES. The systematic review was registered with PROSPERO.

**Results:**

Our combined data set included 29 cases (9 RCVS and 20 PRES). Most cases were women (72.4%) with a mean age of 50.7 years (SD 19.4 years). Most cases were associated with COVID-19 mRNA vaccines (58.6% Moderna, 20.7% Pfizer). Hypertension (37.9%), hyperlipidemia (13.7%), chronic kidney disease (CKD) (10.3%), and end-stage renal disease (6.8%) were common comorbidities. Furthermore, 20.6% (6/29) of cases were on immunosuppression therapy for various reasons. The mean time to symptom onset was 10.49 days after vaccination (SD 18.60), and the mean duration of hospitalization was 7.42 days (SD 5.94). The symptoms reported the most frequently were headache (41.3%), elevated blood pressure (31.0%), and emesis (17.2%). Typical radiographic findings included T2/FLAIR hyperintensities affecting the parieto-occipital lobes, indicative of vasogenic and/or cytotoxic edema.

**Conclusions:**

This study provides a comprehensive analysis of postvaccine RCVS and PRES. Both disease states were seen most often in those with pre-existing risk factors such as female sex, age over 50, hypertension, renal disease, and immunosuppression. Vaccines and their associated immune response may cause endothelial dysfunction leading to cerebral vasospasm and loss of cerebral autoregulation. However, further research is required to understand the underlying pathophysiological mechanisms. Despite the associations found, the absolute risk of these syndromes remains extremely low compared to the immense benefits of vaccination.

## Introduction

Highlights

- The study aimed to provide a comprehensive analysis of reversible cerebral vasoconstriction syndrome (RCVS) and posterior reversible encephalopathy syndrome (PRES) that arise secondary to vaccination through an analysis of Vaccine Adverse Event Reporting System and a systematic review.
- A total of 29 cases were analyzed, with most cases (58.6% Moderna, 20.7% Pfizer) associated with COVID-19 mRNA vaccines. Many cases were women, with a mean age of 50.7 years.
- Common comorbidities included hypertension, hyperlipidemia, chronic kidney disease, and end-stage renal disease. Additionally, 20.6% of the cases were on immunosuppression therapy. Symptoms frequently reported were headache, elevated blood pressure, and emesis, with radiographic findings often showing T2/FLAIR hyperintensities in the parieto-occipital lobes on MRI.
- The risk of postvaccine RCVS and PRES may be higher in those with pre-existing risk factors. Vaccination and its associated inflammation may lead to blood-brain barrier breakdown, endothelial dysfunction leading to loss cerebral autoregulation and vasospasm. However, further research on the exact pathophysiological mechanisms is necessary.
- While the study found associations between certain risk factors (female sex, age over 50, hypertension, renal disease, and immunosuppression) and postvaccine RCVS and PRES, the overall risk remains minimal compared to the vast benefits of vaccination.

Vaccines have revolutionized global health, preventing millions of deaths annually by immunizing against various infectious diseases^1^. However, as with any medical intervention, vaccines can have adverse effects. The Vaccine Adverse Event Reporting System (VAERS) is a national early warning system in the United States that monitors vaccine safety. VAERS provides a platform for healthcare professionals, vaccine manufacturers, and the public to report possible side effects or adverse events (AEs) that may be associated with vaccines^2^. Despite its limitations, such as under-reporting and the potential for bias, VAERS plays an essential role in postmarketing surveillance of vaccine safety and efficacy^2^.

Reversible cerebral vasoconstriction syndrome (RCVS) is characterized by recurrent thunderclap headaches and cerebral vasoconstriction that typically resolves over days to weeks^3^. Posterior reversible encephalopathy syndrome (PRES) (less commonly referred to as reversible posterior leukoencephalopathy syndrome) is a clinical-radiological entity characterized by headaches, seizures, altered consciousness, and visual disturbances associated with typical imaging findings of posterior cerebral white matter edema^4^. PRES and RCVS are believed to have coinciding pathophysiological mechanisms, including cerebral vasospasm and loss of cerebral autoregulation^5^. Although rarely reported, thrombotic phenomena, including stroke and vasospastic disorders, have been reported as an adverse reaction to vaccination^6,7^.

Isolated postvaccine RCVS and PRES cases have been reported; however, no comprehensive analysis of these events has been conducted concerning vaccine administration. This study aims to provide an analysis of RCVS and PRES after vaccination, as reported in the VAERS database as well as a systematic review of the literature. We aim to contribute to our understanding of these rare postvaccine neurological syndromes, including compounding risk factors and outcomes, to guide clinicians in managing these potential AEs.

## Methods

### VAERS database analysis

The VAERS database (https://vaers.hhs.gov/) is a national early warning system that monitors the safety of vaccines in the United States and was searched to identify cases of PRES and RCVS after vaccination^2^. The VAERS database was accessed on 1 August 2023, and no constraints were placed on the reporting year. The search strategy was constructed using the key indexing terms ‘reversible cerebral vasoconstriction syndrome’ and ‘posterior reversible encephalopathy syndrome’. VAERS ID was identified for all cases and extracted in a txt file. Each case was assessed by two reviewers. Cases of RCVS were included in the results if they had a score of ≥3 on the validated RCVS_2_ score for reversible cerebral vasoconstriction syndrome or if there were radiographic findings consistent with the diagnosis. The RCVS_2_ score is a clinical prediction rule used to diagnose RCVS. The RCVS_2_ score is calculated based on several clinical and radiological factors to differentiate RCVS from other conditions like aneurysmal subarachnoid hemorrhage, primary angiitis of the central nervous system, and other causes of thunderclap headache or reversible cerebral vasoconstriction. The score typically incorporates elements such as: (1) internal carotid artery involvement (not affected 0; affected −5), (2) sex (male 0; female +1), (3) vasoconstrictive trigger identified (no 0; yes +3), (4) subarachnoid hemorrhage present on imaging (absent 0; present +1) (5) recurrent or single thunderclap headache (absent 0; present +5). Scores 3–4 had 86% specificity and 10% sensitivity for the diagnosis of RCVS^8^. This study was completed according to the Strengthening the Reporting of Cohort Studies in Surgery (STROCSS) criteria^9^. The research protocol was prospectively registered on the Center for Open Science (OSF) registry.

### Systematic review and search strategy

This systematic review was conducted according to the Preferred Reporting Items for Systematic Reviews and Meta-analysis (PRISMA) and assessing the methodological quality of systematic reviews (AMSTAR) guidelines^10,11^. The study was registered with the International Prospective Register of Systematic Reviews (PROSPERO). A comprehensive literature search of PubMed/MEDLINE, ScienceDirect, Hinari, and Scopus databases was performed to identify vaccination-related RCVS and PRES. The literature search was conducted on 1 August 2023, and no constraints were placed on the reporting year. The search strategy was built around combinations of key terms: (vaccine OR vaccination) AND (Reversible Cerebral Vasoconstriction Syndrome OR RCVS OR Posterior Reversible Encephalopathy Syndrome OR PRES). The specific search string utilized is identified in Table 1. The search was not restricted by age, sex, type of vaccine, or time frame. Indexing tools such as MeSH terms were used in PubMed/MEDLINE. A gray literature search was conducted by reviewing the first 100 results obtained from Google Scholar. Both forward and backward citations were utilized to include relevant articles.

**Table 1:** Search term utilized for each respective database in systematic review.

Database	Search string
PubMed/Medline	(vaccine OR vaccination) AND (“Reversible Cerebral Vasoconstriction Syndrome”[MeSH/Tiab] OR “RCVS” OR “Posterior Reversible Encephalopathy Syndrome”[MeSH/Tiab] OR “PRES”)
Scopus	vaccine OR vaccination AND “reversible cerebral vasoconstriction syndrome” OR RCVS OR “posterior reversible encephalopathy syndrome” OR PRES
ScienceDirect	(vaccine OR vaccination) AND (Reversible Cerebral Vasoconstriction Syndrome OR Posterior Reversible Encephalopathy Syndrome)
Hinari	vaccine OR vaccination AND Reversible Cerebral Vasoconstriction Syndrome OR Posterior Reversible Encephalopathy Syndrome

### Study selection and data extraction

The exported search results were uploaded to Microsoft EndNote X9 and duplicate records were removed. Screening of titles and abstracts was performed independently by two authors using the Rayyan QCRI web application, a systematic review tool that allows for blind screening and collaboration^12^. Discrepancies between reviewers were resolved through discussion, and if necessary, a third reviewer was consulted. Full-text articles identified as potentially eligible were obtained and evaluated for inclusion based on the following criteria: (1) case reports/series and observational studies of patients who developed RCVS or PRES after vaccination, (2) reports available in English. Exclusion criteria included (1) studies not reporting original patient data (i.e. reviews, editorials, commentaries, and letters), (2) reports where RCVS or PRES could not definitively be attributed to vaccination or where another causative factor was present, (3) records published in nonpeer-reviewed journals. Data extraction was performed by two independent reviewers and consistency was cross-checked. The extracted data included the type of vaccine, the time to onset of RCVS or PRES symptoms after vaccination, patient comorbidities demographic information, radiographic findings, clinical manifestations, hospital stay, time to symptom onset, imaging findings, treatment, and outcomes.

### Data characterization and analysis

Descriptive statistics were used to analyze the extracted data. Data were presented as mean±SD and as frequencies and percentages for categorical variables. All statistical analyzes were performed using Python v3.8 with Pandas v1.1.3 and NumPy v1.18.5 libraries for data management and manipulation. Data visualization was completed using the Python library Matplotlib v3.3.2.

### Quality and risk of bias assessment

The quality of the included records within the systematic review was evaluated using the Joanna Briggs Institute (JBI) critical appraisal tool for case reports. The tool consists of eight items addressing different aspects of the methodological quality of case reports, including precise patient demographics, accurate diagnosis, objective measurements of intervention outcomes, and follow-up information. The quality and risk of bias assessment were conducted by two authors; a third author was consulted for any discrepancy between reviewers.

## Results

### VAERS database results

A total of 11 RCVS and 26 PRES diagnoses were registered in the VAERS database. Our analysis yielded a total of 7 RCVS and 18 PRES events based on manual review using the inclusion criteria. Thus, our VAERS data set is made up of 25 cases. Most of the reports were from women (72%), averaging 52.42±18.42 years (range 18–93). In terms of regional distribution, Ohio was the most reported region, with four instances, followed by Florida and Illinois, each with three cases. The onset of symptoms after vaccination was on average, 11.42 days (SD 19.71, range 1–90 days). For those hospitalized, the average duration of hospitalization was 7.42 days (SD 5.94, range 1–25 days). The most reported comorbidities were hypertension (10/25; 40%) and hyperlipidemia (4/25; 16%). Chronic kidney disease (CKD) was observed in 3/25 (12%) cases, and end-stage renal disease (ESRD) in 2/25 (8%) cases. Additionally, 6/10 (24%) individuals were on immunosuppression therapy, including pembrolizumab for metastatic breast cancer, tacrolimus and mycophenolate after a kidney transplant, and voclosporin for lupus nephritis. The symptoms reported the most frequently included headache (10/25; 40%), elevated blood pressure (9/25; 36%), and emesis (5/25; 20%). Regarding the types of vaccine-associated with these events, most were related to the COVID-19 mRNA (Moderna) vaccine, accounting for 15/25 (60%) cases. The COVID-19 mRNA vaccine (Pfizer) was reported in 6/25 (24%) instances, while other vaccines (i.e. pneumococcal, influenza, COVID-19 [Janssen]) comprised the rest. Management was often symptomatic for related symptoms, including control of blood pressure and levetiracetam for managing seizures. Outcomes were only reported in 7/25 (28%) cases and were favorable. Table 2 provides complete clinical details for each case alongside the respective VAERS ID.

**Table 2:** VAERS analysis for cases of RCVS and PRES after vaccination.

VAERS ID	Diagnosis	Age	Sex	Region	Year Reported	Comorbidities	Symptoms	Hospitalization	Vaccine Type	Time to onset	Neurodiagnostic Testing	Treatment	Outcomes
1272049-1	RCVS	41	M	Florida	2021	–	Dizziness, nausea, emesis, weakness, speech impairment	No	COVID-19 mRNA (Pfizer)	4 days	MRI unspecified findings of a CVA	–	–
1040300-1	RCVS	41	M	Connecticut	2021	Hypertension, caffeine consumption (60 oz daily)	Thunderclap headache	10 days	COVID-19 mRNA (Moderna)	5 days	CTH showing SAH. DSA: Evidence of the bilateral PCAs, SCA, and L PICA narrowing.	Nimodipine, GABApentin, and 1 week course of levetiracetam.	–
1071969-1	RCVS	51	M	Wisconsin	2021	Postcoital headaches	Headache, nausea, emesis	4 days	COVID-19 mRNA (Moderna)	4 days	CTH: subarachnoid hemorrhage	–	–
1354795-1	RCVS	75	F	Ohio	2021	Hypertension	Severe headache	No	COVID-19 mRNA (Moderna)	1 day	DSA: near-complete occlusion of L MCA	–	Unspecified improvement.
2309661-1	RCVS	69	F	Illinois	2022	Hyperlipidemia, depression	Thunderclap headache, disorientation, emesis, speech impairment	No	COVID-19 mRNA (Moderna)	8 days	–	Intramuscular Toradol and promethazine.	–
2367879-1	RCVS	56	F	Florida	2022	CVST, IIH, Lyme meningitis, hypertension, hypothyroidism	Thunderclap headache	No	COVID-19 mRNA (Pfizer)	36 days	MRV: Moderate to severe narrowing of the L transverse sinus (chronic)	Acetazolamide	Improved severity of chronic headaches
2596001-1	RCVS	47	F	Texas	2023	Depression (on SSRIs)	Thunderclap headache	3 days	COVID-19 mRNA (Moderna)	48 days	MRI and CTH unspecified results	Verapamil	Improvement in headache
0510187-1	PRES	23	F	Illinois	2013	ESRD, hypertension	Blurry vision and seizures	Yes	Influenza (quadrivalent)	1 day	–	–	–
0742509-1	PRES	–	F	–	2018	–	Cortical blindness, hallucinations, weakness.	Yes	Influenza, quadrivalent.	–	T2/FLAIR hyperintensities involving the parieto-occipital lobes	–	Improvement
0933025-1	PRES	43	F	Arizona	2021	Hyperthyroidism	Thunderclap headache	1 day	COVID-19 mRNA (Moderna)	7 days	MRI/CT: Occipital lobe lesions	Decadron	Improvement in symptoms at 10 days
1060313-1	PRES	73	F	Wyoming	2021	Hypertension	Acute altered mental status, elevated BP (SBP >240 mmHg)	2 days	COVID-19 mRNA (Moderna)	<24 h	MRI: Punctate lesions in the parietal and frontal lobes.	Blood pressure management	
1069614-1	PRES	59	F	West Virginia	2021	Metastatic ovarian breast cancer on chemotherapy (pembrolizumab)	Altered mental status, elevated blood pressure	8 days	COVID-19 mRNA (Moderna)	2 days	MRI: Vasogenic edema affecting the parietal, temporal, occipital, and cerebellar hemispheres.	Blood pressure management (amlodipine), and levetiracetam.	–
1122987-1	PRES	18	M	–	2021	CKD V, Chronic use of tacrolimus due to failed kidney transplant	Generalized tonic-clonic seizures.	7 days	COVID-19 (Janssen)	1 day	MRI: Asymmetric scattered regions of predominantly right-sided subcortical signal abnormality of a posterior predominance. EEG: Bisynchronous rhythmic slow waves with wide distribution (GRDAs)	Levetiracetam	–
1176079-1	PRES	93	M	Virginia	2021	CKD IV, hypertension	Elevated blood pressure (SBP >220 mmHg), seizures, altered mental status	6 days	COVID-19 mRNA (Moderna)	1 day	MRI: T2/FLAIR hyperintensities of the occipital lobes. EEG: Electrographic seizures (unspecified)	Levetiracetam	–
1290132-1	PRES	64	F	Ohio	2021	Necrotizing pancreatitis	Altered mental status, acute hypoxic and hypercapnic respiratory failure, splenic vein thrombosis	25 days	COVID-19 mRNA (Pfizer)	11 days	MRI: PRES (unspecified)	–	–
1330290-1	PRES	33	F	Ohio	2021	Kidney transplant on tacrolimus	Encephalopathy, tachycardia, elevated blood pressure	8 days	COVID-19 (Janssen)	6 days	MRI: PRES (unspecified)	–	–
1396565-1	PRES	66	F	Ohio	2021	Scleroderma (mycophenolate mofetil 1000 mg twice daily)	Comatose	3 days	COVID-19 mRNA (Moderna)	11 days	–	–	–
1430482-1	PRES	53	F	Texas	2021	Obesity, macrocytic anemia, CKD, stroke, pulmonary embolus	Headache, nausea, emesis, elevated blood pressure (SBP >250 mmHg)	3 days	COVID-19 mRNA (Moderna)	3 days	MRI: T2/FLAIR hyperintensities involving the bilateral frontal, parietal, and occipital lobes.	–	–
1501696-1	Severe pre-eclampsia + PRES	33	F	Delaware	2021	Infertility	Severe hypertension, proteinuria, cortical blindness, hallucinations	8 days	COVID-19 mRNA (Pfizer)	10 days	–	–	–
1757268-1	PRES	44	F	North Carolina	2021	Substance use disorder, hypertension, type 2 diabetes mellitus, hyperlipidemia, vitamin D deficiency	Elevated blood pressure (SBP >220 mmHg), seizures, altered mental status	10 days	COVID-19 mRNA (Moderna)	4 days	MRI: Vasogenic edema of the occipital lobes	Amlodipine 10 mg, lisinopril 10 mg, hydrochlorothiazide 12.5 mg and carvedilol 3.125 mg twice daily	–
2043972-1	PRES	48	M	Wisconsin	2022	ESRD, immunosuppression (tacrolimus, mycophenolate), hypertension	Flu-like symptoms, shortness of breath, hyperpyrexia (102.5F), generalized tonic-clonic seizure	6 days	COVID-19 mRNA (Moderna)	1 day	MRI: Unspecified.	–	Oxygen-dependent with significant residual symptoms.
2211495-1	PRES	33	M	West Virginia	2022	Hypertension	shakiness, lightheadedness, generalized weakness, difficulty breathing, possible seizures, and headache	4 days	COVID-19 mRNA (Pfizer)	10 days	CTH: Small focal areas of low attenuation involving the bilateral occipital lobes, greater than left. R frontal lobe SAH. EEG: No epileptiform discharges.	–	–
2237513-1	PRES	50	F	Illinois	2022	Lupus nephritis (on voclosporin)	Hyperpyrexia (101.8F), emesis, headache, blurred vision, elevated blood pressure (SBP >200 mmHg)	7 days	COVID-19 mRNA (Moderna)	~90 days	CT/MRI: PRES (unspecified)	–	–
2315645-1	PRES	80	F	Florida	2022	Hyperlipidemia, coronary artery disease, dementia	Aphasia	5 days	COVID-19 mRNA (Moderna)	1 day	MRI: PRES (unspecified)	–	–
2527853-1	PRES	65	F	Puerto Rico	2022	Hyperlipidemia	Headache, paresthesia, weakness, elevated blood pressure	21 days	COVID-19 mRNA (Pfizer) + pneumococcal (Prevnar 13)	8 days	PRES (unspecified)	–	Improvement in symptoms at 3 weeks.

CKD, Chronic kidney disease; COVID-19, Coronavirus disease 2019; CTH, Computerized tomography head; CVA, Cerebrovascular accident; CVST, Cerebral venous sinus thrombosis; DSA, Digital subtraction angiography; EEG, Electroencephalogram; ESRD, End-stage renal disease; GRDA, Generalized rhythmic delta activity; MCA, Middle cerebral artery; MRA, Magnetic resonance angiography; PCA, Posterior cerebral artery; PICA, Posterior inferior cerebellar artery; PRES, Posterior reversible encephalopathy syndrome; RCVS, Reversible cerebral vasoconstriction syndrome; SAH, Subarachnoid hemorrhage; SCA, Superior cerebellar artery; SSRI, Selective serotonin reuptake inhibitor.

### Systematic review results

**Figure 1:**
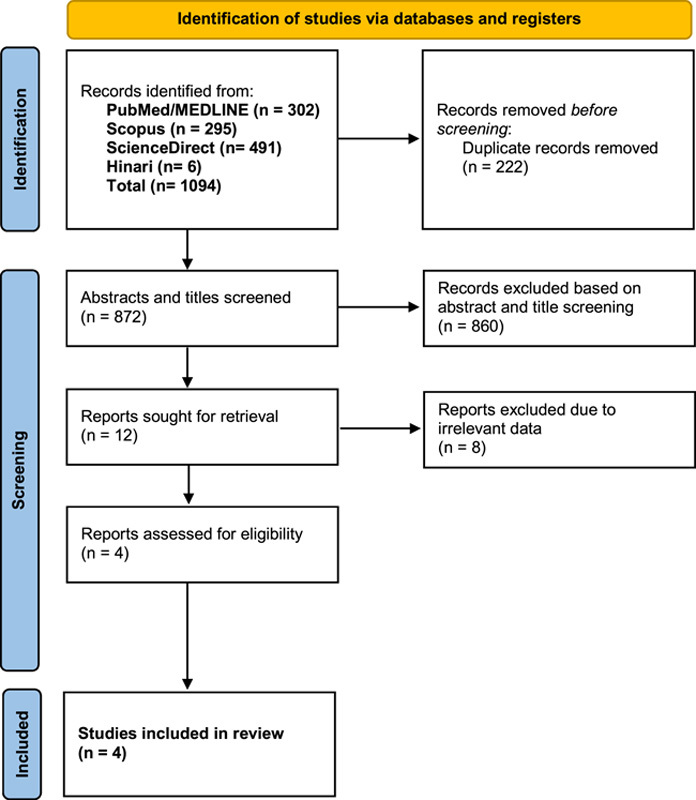
PRISMA 2020 flow diagram for new systematic reviews which included searches of databases and registers only.

A total of 1094 records were identified from four electronic databases in this systematic review. A total of 860 records procured through the search strategy were excluded at the initial abstract and title screening stage. The reason for the removal was due to irrelevant data to the study protocol. A total of 4 cases were included in the results^13–16^. A PRISMA flow diagram is provided in Figure 1. Two cases of RCVS and PRES were identified. The average was 40.5 years, and 3/4 cases were reported in women. Two instances of RCVS were reported after administering the mRNA COVID-19 vaccine. A case of PRES was reported secondary to the measles vaccine and another from the mRNA COVID-19 vaccine. 3/4 cases had a symptom onset within 24 h after receiving their vaccine, whereas case no. 4 reported RCVS symptoms, including blurry vision and a focal headache 18 days after vaccination. Common risk factors included a history of RCVS, recreational drug use, alcohol and smoking, and hypertension. Common findings reported in people with PRES include T2/FLAIR hyperintensities on MRI indicative of vasogenic edema. The radiographic findings in RCVS also included vasogenic edema within the posterior circulation and focal stenosis of the posterior cerebral artery (P1 segment). Additionally, case no. 3 had a history of RCVS reported two years before his current presentation and had radiographic findings of basilar stenosis with a ‘beads-on-a-string’ appearance. Treatment included levetiracetam in two cases. Case no. 2 was also treated with three days of pulse-dose steroids. The management of RCVS had a short course of losartan in anticipation of a subsequent dose of the COVID-19 mRNA vaccine and oral nimodipine for cerebral vasospasm. 75% of cases showed favorable outcomes with resolution of symptoms; however, case no. 1 continued to exhibit cognitive deficits at 18 months of follow-up. All included cases are shown in Table 3.

**Table 3:** Results of the systematic review of cases of PRES and RCVS after vaccination.

Case no.	Diagnosis	Age	Sex	Risk factors	Symptoms	Vaccine type	Time to onset	Imaging findings	Treatment	Outcomes
1	PRES	76	F	Hypertension, alcohol use disorder, shingles	Confusion, blurry vision, unsteady intermittent gait	COVID-19 mRNA (Moderna)	<24 h	T2/FLAIR hyperintensities in the parieto-occipital lobes	levetiracetam 750 mg twice daily	Residual cognitive decline 18 months later
2	PRES	18	F	None	Diffuse pain, pruritus, weakness of the hand, dysthesia	Measles	8 h	T2/FLAIR hyperintensities in the occipital lobes	1000 mg steroids for 3 days. MRA showed vasoconstriction of the posterior cerebral arteries	Improvement in T2/FLAIR hyperintensities and posterior circulation vasoconstriction after 3 days of treatment
3	RCVS	30	M	History of RCVS, Bipolar disorder	Thunderclap headache	COVID-19 mRNA (Pfizer)	12 h	Normal CT head	Losartan 50 mg for 2 weeks	No symptoms at 2-week follow-up
4	RCVS	38	F	Migraine without aura, 10-pack smoking history	Sudden-onset blurred vision bilaterally followed by a focal headache over the right occipital projection	COVID-19 mRNA (Moderna)	18 days	MRI showed an ischemic stroke on the territory of the right posterior cerebral artery. MRA shows discontinuation of the right P1 segment	Levetiracetam (1000 mg/d) and nimodipine (90 mg/d) for 5 weeks	Normal flow in both PCAs was documented on CTA after 7 days of nimodipine, suggesting vasospasm

COVID-19, Coronavirus disease 2019; MRA, Magnetic resonance angiography; PRES, Posterior reversible encephalopathy syndrome; RCVS, Reversible cerebral vasoconstriction syndrome.

### Quality and risk of bias assessment

The Joanna Briggs Institute critical appraisal tool for case reports was used to assess the risk of bias in reported cases within the systematic review. Overall, a low risk of bias was observed in the four cases. Table 4 provides a checklist for all included records^13–16^.

**Table 4:** Joanna Briggs Institute critical appraisal and risk of bias results for included records.

Reference	Q1	Q2	Q3	Q4	Q5	Q6	Q7	Q8	Overall	Risk
McCullough *et al*.^16^	Y	Y	Y	Y	Y	Y	Y	Y	8	Low
Hamano *et al*.^14^	Y	Y	Y	Y	Y	N	Y	Y	7	Low
Lund *et al*.^15^	Y	Y	Y	Y	Y	Y	Y	Y	8	Low
Finsterer^13^	Y	Y	Y	Y	Y	Y	Y	Y	8	Low

### Combined analysis

Our combined data set included a total of 29 patients [9 (31.0%) RCVS and 20 (69%) PRES], with the majority being female (*n*=21, 72.4%) compared to males (*n*=8, 27.6%). The age of the patients ranged from 18 to 93 years, with a mean age of 50.7 years (SD 19.4 years; range 18–93). Geographically, the regions that were the most frequently reported were Florida, Ohio, and Illinois. Other areas represented in the data included Wisconsin, Texas, West Virginia, Connecticut, Arizona, Wyoming, Virginia, Delaware, North Carolina, and Puerto Rico. Among the patients, common comorbidities included hypertension (*n*=11, 37.9%), and hyperlipidemia (*n*=4, 13.7%). CKD was observed in 3/3 (10.3%) cases, and end-stage renal disease (ESRD) in 2/3 (6.8%) cases. Furthermore, 6/29 (20.6%) individuals were on immunosuppressive therapy for various reasons, including kidney transplants, breast cancer, and lupus nephritis. The most common CTH finding was hypodensities within the posterior occipital regions of the brain. And MRI often revealed T2/FLAIR hyperintensities consistent with vasogenic edema within the parietal and occipital lobes.

**Figure 2:**
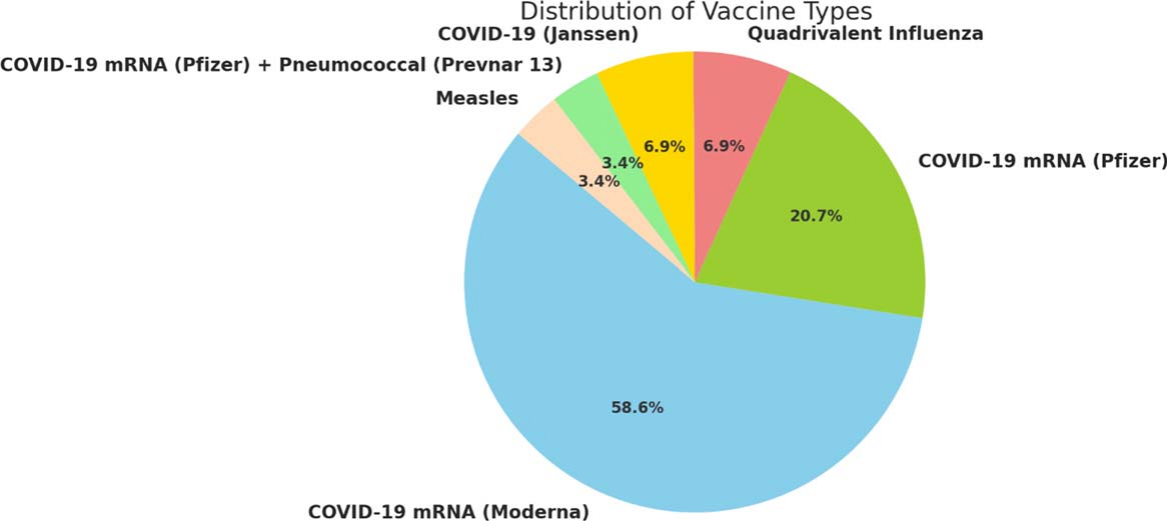
Graphical breakdown of the 29 vaccine related cases of RCVS and PRES.

The most frequently reported symptom was headache (*n*=12, 41.3%), followed by elevated blood pressure (*n*=9, 31.0%) and emesis (*n*=5, 17.2%). Note that patients often presented multiple symptoms. The onset of symptoms after vaccination was ~10.49 days (SD=18.60). The mean duration of hospitalization among patients was ~7.42 days, although this varied widely between individuals, with a SD of 5.94 days. COVID-19 mRNA vaccines from Moderna (*n*=17, 58.6%) and Pfizer (*n*=6, 20.7%) were the most frequent in our data set. Other vaccines included the quadrivalent influenza vaccine (*n*=2, 6.8%), the Janssen COVID-19 vaccine from Janssen (*n*=2, 6.8%), a combination of the Pfizer COVID-19 mRNA vaccine and the pneumococcal vaccine Prevnar 13 (*n*=1, 3.4%), and the measles vaccine (*n*=1, 3.4%). Figure 2 provides a visual representation of the vaccines involved. Management was often symptomatic for related symptoms, including control of blood pressure and levetiracetam for managing seizures. Only 10/29 (34.5%) cases reported results, 8/10 (80%) of which were favorable.

## Discussion

Our analysis of the VAERS database and systematic review represents the most comprehensive evaluation of RCVS and PRES after vaccination. We included 29 cases (9 RCVS and 20 PRES), most of which were reported in women (72.4%), and those with pre-existing risk factors, including hypertension, renal disease, and immunosuppressive therapy. The COVID-19 mRNA vaccines were most often implicated. Understanding the pathophysiology of RCVS and PRES is essential in understanding how vaccines might contribute to their development. RCVS and PRES are understood as disorders of cerebral autoregulation and vasospasm. In RCVS, there is a transient alteration in the tone of the cerebral arteries, leading to vasoconstriction that can fluctuate and eventually reverses completely^3^. PRES is believed to result from the failure of the cerebral autoregulation mechanism in response to acute changes in blood pressure, resulting in hyperperfusion and endothelial dysfunction, leading to vasogenic edema^17^. The endothelium of cerebral vessels produces several vasoactive substances including nitric oxide and eicosanoids, which help regulate cerebral blood flow^18^. Vaccines, particularly COVID-19 mRNA vaccines, have been proposed to induce a robust immune response that could lead to systemic inflammation and endothelial dysfunction^19^. Thus, disrupting cerebral autoregulation and promoting vasospasm, leading to conditions such as RCVS and PRES. Furthermore, the SARS-CoV-2 spike protein has been suggested to injure endothelial cells directly, and the spike protein produced in response to mRNA vaccines could have similar effects^20,21^.

In our study, the mean age of the patients was 50.7 years, and common comorbidities included hypertension, hyperlipidemia, immunosuppression, and CKD. These findings are consistent with known risk factors for RCVS and PRES. The typical imaging finding in RCVS includes focal stenosis or narrowing of the cerebrovasculature, as seen on CTA, and digital subtraction angiography. The radiographic findings of PRES also include vasogenic edema on magnetic resonance imaging, as this was our cohort’s most prevalent radiographic finding^22,23^. Careful monitoring and management of these conditions in the postvaccination period may be needed, especially in patients with compounding risk factors as seen in our cohort. Our study’s clinical presentations of RCVS and PRES were typical, with the most frequently reported symptoms being headache, elevated blood pressure, and emesis. The onset of symptoms after vaccination was ~10.49 days on average, indicating a potential window for early intervention and treatment. The management strategies in our study were largely symptomatic, focusing on controlling blood pressure and managing seizures. About a third of the cases reported favorable outcomes, which may reflect the nature of these conditions and the challenges associated with their management.

Our study has several limitations, including possible under-reporting and reporting bias in the VAERS database. A ʻstimulatedʼ reporting bias may have occurred due to the COVID-19 pandemic. Thus, many cases of non-mRNA vaccine-related AEs prior to the COVID-19 pandemic may have been underreported. As an observational study, we cannot establish a causal relationship between vaccination and RCVS/PRES. Despite these limitations, our study provides important information on these rare postvaccine neurological syndromes, helping inform clinicians and guide future research. The study also underscores the importance of ongoing surveillance and more research to understand the potential relationship between vaccination and the development of these rare neurological syndromes. Future studies are needed to validate our findings in larger cohorts and to elucidate the underlying pathophysiological mechanisms. Such studies can evaluate mRNA vaccines and causality with vasospasm and blood-brain barrier dysfunction. Guiding the development of strategies for preventing, detecting, and managing these potential postvaccine AEs. Despite the associations identified, it is crucial to emphasize that the absolute risk of developing these syndromes after vaccination remains extremely low. The findings of this study should not discourage the public from getting vaccinated but should serve to inform healthcare professionals for better recognition, reporting, and management of these rare events.

## Conclusions

We identified 29 cases of RCVS and PRES after vaccination, most of which were associated with COVID-19 mRNA vaccines. Although these events are rare, our study highlights the importance of maintaining vigilance for these potential neurological syndromes in the postvaccination period, particularly in patients with pre-existing risk factors such as female sex, age over 50, underlying hypertension, immunosuppressant usage, and renal disease. Our findings suggest that the immune response triggered by vaccines, particularly mRNA-based COVID-19 vaccines, could theoretically disrupt cerebral autoregulation and promote vasospasm, leading to conditions such as RCVS and PRES. However, establishing a causal relationship will require further investigation.

## Ethical approval

The Vaccine Adverse Event Reporting System (VAERS) is under the jurisdiction and ethical approval by the U.S. Department of Health and Human Services (HHS). VAERS is co-managed by the Centers for Disease Control and Prevention (CDC) and the U.S. Food and Drug Administration (FDA). Ethical approval was not required for this research because no patient records were accessed.

## Consent

The VAERS complies with all United States Government security standards and protections concerning health information. Use of the VAERS database does not require approval by an institutional review board or informed consent.

## Sources of funding

No internal or external funding was received for this manuscript.

## Author contribution

B.S.S.: conceptualization, data curation, formal analysis, methodology, project administration, resources, software, validation, visualization, writing – original draft, writing – review and editing; T.F.: writing – original draft; V.K. and M.A.G.D.: conceptualization, writing – original draft, writing – review and editing.

## Conflicts of interest disclosure

On behalf of all authors the corresponding author states that there are no conflicts of interest.

## Research registration unique identifying number (UIN)

Open Science Framework https://doi.org/10.17605/OSF.IO/KPMDE.

## Guarantor

Bahadar S. Srichawla.

## Data availability statement

Data is available upon reasonable request from the Editor-In-Chief.

## Provenance and peer review

Not commissioned, externally peer-reviewed.
